# Supplementary material for: Fetal Brain-Derived Exosomal miRNAs from Maternal Blood: Potential Diagnostic Biomarkers for Fetal Alcohol Spectrum Disorders (FASDs)
Source: Int J Mol Sci. 2024 May 27;25(11):5826. doi: 10.3390/ijms25115826 (PMC11172088; doi:10.3390/ijms25115826)
Supplement: Supplementary file 1 [file ijms-25-05826-s001.zip › ijms-2963649-supplementary.pdf]

Supplemental Data

Table S1. Anatomical and molecular targets of miRNAs represented in the Neuralogical Development and Disease Array used in this study.

Name of miRNA	Target	Action	Citation
**Autistic Disorders**			
miR-106b-5p	IRF1	Downregulation	[S1]
miR-128	Basal Ganglia	Downregulation	[S2]
miR-132-3p	Neuronal Septum	Downregulation	[S3]
miR-140-5p	NF-κB	Downregulation	[S4]
miR-146b-5p	IRAK1	Downregulation	[S5]
miR-148b-3p	GLUT1	Downregulation	[S6]
miR-15a-5p	Lipid Metabolism	Downregulation	[S7]
miR-15b-5p	Wnt Signaling	Upregulation	[S8]
miR-181d	Cerebellum	Upregulation	[S9]
miR-193b-3p	Wnt Signaling	Upregulation	[S10]
miR-212-3p	Amygdala	Downregulation	[S11]
miR-27a-3p	Adipogenesis	Downregulation	[S12]
miR-320a	Hedgehog Signaling Pathway	Downregulation	[S13]
miR-381-3p	FoxO Pathway	Downregulation	[S14]
miR-431-5p	XIAP	Downregulation	[S15]
miR-432-5p	Glutamatergic Synapse	Upregulation	[S16]
miR-484	Ventricular surface	Downregulation	[S17]
miR-539-5p	Cerebellar Cortex	Upregulation	[S18]
miR-652-3p	B Cell Receptor Regulation	Upregulation	[S19]
amiR-7-5p	Dopaminergic Differentiation	Downregulation	[S20]
miR-93-5p	Human Growth Factor	Downregulation	[S21]
miR-95	Cerebellar Cortex	Upregulation	[S22]
**Schizophrenia**			

let-7d-5p	Wnt Signaling	Upregulation	[S23]
let-7e-5p	Osteoblast Differentiation	Upregulation	[S24]
miR-105-5p	FAK/Akt signaling pathway	Downregulation	[S25]
miR-106b-5p	Caspase-3	Upregulation	[S26]
miR-107	LTP Pathway	Upregulation	[S27]
miR-126-5p	Cytokines IL-1β	Downregulation	[S28]
miR-128	PPAR-γ	Downregulation	[S29]
miR-130a-3p	Ly6Clo-prone Macrophages	Upregulation	[S30]
miR-138-5p	Hippocampal Interneurons	Downregulation	[S31]
miR-152	T cell Proliferation	Upregulation	[S32]
miR-15a-5p	Chemokine ligand	Downregulation	[S33]
miR-15b-5p	BDNF	Downregulation	[S27, S34]
miR-181a-5p	GluA2	Downregulation	[S35]
miR-195-5p	TGF-β1	Downregulation	[S36]
miR-20a-5p	RhoA pathway	Upregulation	[S37]
miR-212-3p	Caspase-3	Upregulation	[S38]
miR-24-3p	IL-6	Downregulation	[S39]
miR-26b-5p	Casepase-3	Upregulation	[S40]
miR-27-3p	Wnt/ß-catenin signaling	Upregulation	[S41]
miR-29a-3p	Prefrontal Cortex	Upregulation	[S42]
miR-29b-3p	AMPK pathway	Downregulation	[S43]
miR-29c-3p	FOXP1 Pathway	Downregulation	[S44]
miR-302a-5p	NF-κB	Downregulation	[S45]
miR-302b-5p	IRF-5	Downregulation	[S46]
miR-30d-5p	Integrin α5	Downregulation	[S47]
miR-338-3p	E-cadherin	Upregulation	[S48]
miR-346	FOXA1	Downregulation	[S49]
miR-381-3p	TNF Apoptosis	Downregulation	[S50]
miR-409-3p	Akt signaling	Downregulation	[S51]

miR-455-5p	TFK-1	Downregulation	[S52]
miR-484	VEGF-A	Downregulation	[S53]
miR-485-5p	FLOT2	Downregulation	[S54]
miR-487a	TGF-β	Upregulation	[S55]
miR-489	NF-Kb	Downregulation	[S56]
miR-499a-5p	CK1α	Downregulation	[S57]
miR-512-3p	Caspase-3	Downregulation	[S58]
miR-518b	Trophoblast Proliferation	Upregulation	[S59]
miR-7-5p	IRS2	Downregulation	[S60]
miR-9-3p	FOXG1	Downregulation	[S61]
miR-92a-3p	β-catenin Signaling Pathway	Upregulation	[S62]
**Anxiety Disorders**			
miR-128	PPAR-γ	Downregulation	[S29, S63]
miR-485-3p	TNF-α	Downregulation	[S64]
miR-509-3p	CDK2	Downregulation	[S65]
**Tourette's Syndrome**			
miR-24-3p	Caspase-8	Downregulation	[S66]
**Alzheimer's Disease**			
let-7b-5p	HK2	Downregulation	[S67]
let-7c	IL-6	Downregulation	[S68]
let-7d-5p	p53 Signaling Pathway	Downregulation	[S69]
let-7e-5p	GF-β Signaling Pathway	Upregulation	[S70]
let-7i-5p	TGFβ-1	Upregulation	[S71]
miR-101-3p	PI3K Signaling Pathways	Downregulation	[S72]
miR-106b-5p	Rho/ROCK1 Pathway	Upregulation	[S73]
miR-107	GLUT4	Downregulation	[S74]
miR-128	FOXO4	Downregulation	[S75]
miR-139-5p	cAMP Signaling Pathway	Upregulation	[S76]
miR-146a-5p	IL-1 receptor (TIR) signaling	Downregulation	[S77]

miR-15a-5p	MAPK-ERK pathway	Upregulation	[S33, S78]
miR-150-5p	Caspase-8	Upregulation	[S79]
miR-151a-3p	TGF-β1	Upregulation	[S80]
miR-181a-5p	NF-κB	Downregulation	[S81]
miR-19b-3p	IGF-1	Upregulation	[S82]
miR-20a-5p	PI3K/Akt signaling pathway	Downregulation	[S83]
miR-22-3p	Src Signaling	Downregulation	[S84]
miR-24-3p	Neuronal Apoptosis	Upregulation	[S85]
miR-26b-5p	EMT Pathway	Downregulation	[S86]
miR-27a-3p	FoxO Signaling	Downregulation	[S87]
miR-28-5p	Cyclin D1	Downregulation	[S88]
miR-298	NF-κB	Downregulation	[S89]
miR-29a-3p	Wnt/β-Catenin Signaling	Downregulation	[S90]
miR-29b-3p	IL-1β	Upregulation	[S43, S91]
miR-29c-3p	FOXP1	Downregulation	[S44, S92]
miR-30d-5p	Integrin α5	Downregulation	[S47, S93]
miR-320a	MafF/Nrf2 signal pathway	Downregulation	[S94]
miR-328	TGF-β3	Upregulation	[S95]
miR-346	Wnt/β-Catenin Pathway	Upregulation	[S96]
miR-34a-5p	Wnt Signaling Pathway	Downregulation	[S97]
miR-376b-3p	FOXP2	Downregulation	[S98]
miR-433	PI3K/Akt Signaling Pathway	Downregulation	[S99]
miR-485-5p	WBP2/Wnt Signaling Pathway	Downregulation	[S100]
miR-488-3p	p53 Pathway	Upregulation	[S101]
miR-511	Hedgehog Signaling Pathway	Upregulation	[S102]
miR-9-5p	β-catenin Signaling Pathway	Upregulation	[S103]
miR-92a-3p	β-catenin Signaling Pathway	Upregulation	[S62, S104]
miR-93-5p	PD-L1/CCND1 Signaling Pathway	Downregulation	[S105]
miR-98-5p	IL-6	Downregulation	[S106]

**Prion Diseases**			
let-7b-5p	HK2	Downregulation	[S67, S107]
miR-128	PPAR-γ	Downregulation	[S29, S108]
miR-139-5p	cAMP Signaling Pathway	Upregulation	[S76, S109]
miR-146a-5p	IL-1 receptor (TIR) signaling	Downregulation	[S77, S110]
miR-191-5p	IRS1/AKT Pathway	Downregulation	[S111]
miR-203a	Caspase 9,3	Upregulation	[S112]
miR-320a	MafF/Nrf2 Pathway	Downregulation	[S94, S114]
miR-337-3p	Wnt/β-catenin Signaling Pathway	Downregulation	[S114]
miR-338-3p	MAPK Signaling Pathway	Downregulation	[S115]
miR-339-5p	Inflammatory Cytokines	Downregulation	[S116]
miR-342-3p	Wnt Pathway	Downregulation	[S117]
**Huntington's Disease**			
miR-124-3p	GSK-3β/β-catenin Signaling Pathway	Downregulation	[S118]
miR-132-3p	NF-κB Signaling Pathway	Downregulation	[S119]
miR-135b-5p	Hippo Signaling Pathway	Upregulation	[S120]
miR-29a-3p	TNFα-induced Adhesion	Downregulation	[S121]
miR-29b-3p	IL-1β	Upregulation	[S43, S122]
miR-9-5p	Nrf2/Keap1 Signaling	Upregulation	[S123]
miR-9-3p	FOXG1 SAP97, Synaptic plastisity	Downregulation Downregulation	[S61, S124]
**Parkinson's Disease**			
miR-133b	PI3K/Akt Signaling Pathway	Upregulation	[S125]
mir-433	PI3K/Akt Signaling Pathway	Downregulation	[S99, S126]
miR-7-5p	IRS2	Downregulation	[S60, S127]
**Spinocerebellar Ataxia 1**			
miR-101-3p	PI3K Sgnaling Pathways	Downregulation	[S72, S128]
miR-130-3p	TGF-β1	Downregulation	[S129]
miR-19b-3p	PI3K/Akt/mTOR Pathway	Upregulation	[S130]

Suplemental References

S1. Shi Y, Zhang B, Zhu J, Huang W, Han B, Wang Q, Qi C, Wang M, Liu F. miR-106b-5p Inhibits IRF1/IFN-β Signaling to Promote M2 Macrophage Polarization of Glioblastoma. *Onco Targets Ther*. **2020** Jul 30;13:7479-7492.

S2. Shi Z, Piccus Z, Zhang X, Yang H, Jarrell H, Ding Y, Teng Z, Tchernichovski O, Li X. miR-9 regulates basal ganglia-dependent developmental vocal learning and adult vocal performance in songbirds. *Elife*. **2018** Jan 18;7:e29087.

S3. Bludau A, Schwartz U, Zeitler DM, Royer M, Meister G, Neumann ID, Menon R. Functional involvement of septal miR-132 in extinction and oxytocin-mediated reversal of social fear. *Mol Psychiatry*. **2023** Nov 8

S4. Xiao X, Ai R, Tian Y, Mi N, Cheng L, Qian N, Zhu X. Study on the Mechanism of Action of MicroRNA-140-5p in the Treatment of Autism by Regulating the Nuclear Factor Kappa B Signaling Pathway. *Indian J Pharm Sci* **2021**:83(5) spl issue "133-139".

S5. Wu X, Li W, Zheng Y. Recent Progress on Relevant microRNAs in Autism Spectrum Disorders. *Int J Mol Sci*. **2020** Aug 17;21(16):5904.

S6. Jing XL, Xing AY, Bai H, Wu L. miRNA-148b-3p Influences Glucose Metabolism of Offspring with Maternal Cholestasis by Regulating GLUT1 Expression in Placental Trophoblast Cells. *Sichuan Da Xue Xue Bao Yi Xue Ban, Journal of Sichuan University.* **2019** May;50(3):328-333, Medicine edition

S7. Ni Y, Yang Y, Ran J, Zhang L, Yao M, Liu Z, Zhang L. miR-15a-5p inhibits metastasis and lipid metabolism by suppressing histone acetylation in lung cancer. *Free Radic Biol Med*. **2020** Dec;161:150-162.

S8. Hosokawa R, Yoshino Y, Funahashi Y, Horiuchi F, Iga JI, Ueno SI. MiR-15b-5p Expression in the Peripheral Blood: A Potential Diagnostic Biomarker of Autism Spectrum Disorder. *Brain Sci*. **2022** Dec 22;13(1):27.

S9. Tonacci A, Bagnato G, Pandolfo G, Billeci L, Sansone F, Conte R, Gangemi S. MicroRNA Cross-Involvement in Autism Spectrum Disorders and Atopic Dermatitis: A Literature Review. *J Clin Med*. **2019** Jan 14;8(1):88.

S10. Shen L, Lin Y, Sun Z, Yuan X, Chen L, Shen B. Knowledge-Guided Bioinformatics Model for Identifying Autism Spectrum Disorder Diagnostic MicroRNA Biomarkers. *Sci Rep*. **2016** Dec 21;6:39663.

S11. Cava C, Manna I, Gambardella A, Bertoli G, Castiglioni I. Potential Role of miRNAs as Theranostic Biomarkers of Epilepsy. *Mol Ther Nucleic Acids*. **2018** Dec 7;13:275-290.

S12. Wu H, Pula T, Tews D, Amri EZ, Debatin KM, Wabitsch M, Fischer-Posovszky P, Roos J. microRNA-27a-3p but Not -5p Is a Crucial Mediator of Human Adipogenesis. *Cells*. **2021** Nov 17;10(11):3205.

S13. Vasu M, Anitha A, Thanseem I, Suzuki K, Yamada K, Takahashi T, Wakuda T, Iwata K, Tsujii M, Sugiyama T, Mori N. Serum microRNA profiles in children with autism. *Mol Autism*. **2014** Jul 30;5:40.

S14. Wang Z, Lu T, Li X, et al. Altered Expression of Brain-specific Autism-Associated miRNAs in the Han Chinese Population. *Frontiers in Genetics*. **2022** ;13:865881.

S15. Wang Y, Zhang K, Yuan X, Xu N, Zhao S, Hou L, Yang L, Zhang N. miR-431-5p regulates cell proliferation and apoptosis in fibroblast-like synoviocytes in rheumatoid arthritis by targeting XIAP. *Arthritis Res Ther*. **2020** Oct 6;22(1):231.

S16. Honorato-Mauer J, Xavier G, Ota VK, et al. Alterations in microRNA of extracellular vesicles associated with major depression, attention-deficit/hyperactivity and anxiety disorders in adolescents. *Transl Psychiatry*. **2023**;13(1):47. Published 2023 Feb 6. doi:10.1038/s41398-023-02326-4

S17. Fujitani M, Zhang S, Fujiki R, Fujihara Y, Yamashita T. A chromosome 16p13.11 microduplication causes hyperactivity through dysregulation of miR-484/protocadherin-19 signaling. *Mol Psychiatry*. **2017** Mar;22(3):364-374.

S18. Li J, Xu X, Liu J, Zhang S, Tan X, Li Z, Zhang J, Wang Z. Decoding microRNAs in autism spectrum disorder. *Mol Ther Nucleic Acids*. **2022** Nov 7;30:535-546.

S19. Nuzziello N, Craig F, Simone M, Consiglio A, Licciulli F, Margari L, Grillo G, Liuni S, Liguori M. Integrated Analysis of microRNA and mRNA Expression Profiles: An Attempt to Disentangle the Complex Interaction Network in Attention Deficit Hyperactivity Disorder. *Brain Sci*. **2019** Oct 22;9(10):288.

S20. Kim JY, Kim W, Lee KH. The role of microRNAs in the molecular link between circadian rhythm and autism spectrum disorder. *Anim Cells Syst* (Seoul). **2023** Feb 23;27(1):38-52.

S21. Zhou M, Hou Y, Wu J, Li G, Cao P, Chen W, Hu L, Gan D. miR-93-5p promotes insulin resistance to regulate type 2 diabetes progression in HepG2 cells by targeting HGF. *Mol Med Rep*. **2021** May;23(5):329.

S22. Mansur, F. A., Raman, N. F. A., Rahman, H. A., & Mohd Manzor, N. F. Mechanism of Autism Spectrum Disorder and The Involvement of microRNA. *Malaysian Journal of Science Health & Technology*, **2021**, 6(Special Issue). https://doi.org/10.33102/mjosht.v6iSpecial Issue.120

S23. Popa N, Boyer F, Jaouen F, Belzeaux R, Gascon E. Social Isolation and Enrichment Induce Unique miRNA Signatures in the Prefrontal Cortex and Behavioral Changes in Mice. *iScience*. **2020** Nov 10;23(12):101790.

S24. Wang C, Liu S, Li J, Cheng Y, Wang Z, Feng T, Lu G, Wang S, Song J, Xia P, Hao L. Biological Functions of Let-7e-5p in Promoting the Differentiation of MC3T3-E1 Cells. *Front Cell Dev Biol*. **2021** Sep 8;9:671170.

S25. He B, Zhang K, Han X, Su C, Zhao J, Wang G, Wang G, Zhang L, Hu W. Extracellular Vesicle-Derived miR-105-5p Promotes Malignant Phenotypes of Esophageal Squamous Cell Carcinoma by Targeting SPARCL1 via FAK/AKT Signaling Pathway. *Front Genet*. **2022** Mar 3;13:819699.

S26. Xiang W, He J, Huang C, Chen L, Tao D, Wu X, Wang M, Luo G, Xiao X, Zeng F, Jiang G. miR-106b-5p targets tumor suppressor gene SETD2 to inactive its function in clear cell renal cell carcinoma. *Oncotarget*. **2015** Feb 28;6(6):4066-79.

S27. Beveridge NJ, Gardiner E, Carroll AP, Tooney PA, Cairns MJ. Schizophrenia is associated with an increase in cortical microRNA biogenesis. *Mol Psychiatry*. **2010** Dec;15(12):1176-89.

S28. Pan J, Qu M, Li Y, Wang L, Zhang L, Wang Y, Tang Y, Tian HL, Zhang Z, Yang GY. MicroRNA-126-3p/-5p Overexpression Attenuates Blood-Brain Barrier Disruption in a Mouse Model of Middle Cerebral Artery Occlusion. *Stroke*. **2020** Feb;51(2):619-627.

S29. Budi HS, Younus LA, Lafta MH, Parveen S, Mohammad HJ, Al-Qaim ZH, Jawad MA, Parra RMR, Mustafa YF, Alhachami FR, Karampoor S, Mirzaei R. The role of miR-128 in cancer development, prevention, drug resistance, and immunotherapy. *Front Oncol*. **2023** Jan 19;12:1067974.

S30. Liu L, Wang P, Wang YS, Zhang YN, Li C, Yang ZY, Liu ZH, Zhan TZ, Xu J, Xia CM. MiR-130a-3p Alleviates Liver Fibrosis by Suppressing HSCs Activation and Skewing Macrophage to Ly6Clo Phenotype. Front Immunol. 2021 Aug 5;12:696069.

S31. Daswani R, Gilardi C, Soutschek M, Nanda P, Weiss K, Bicker S, Fiore R, Dieterich C, Germain PL, Winterer J, Schratt G. MicroRNA-138 controls hippocampal interneuron function and short-term memory in mice. Elife. 2022 Mar 15;11:e74056.

S32. Wang Y., Wang D., Xie G., Yin Y., Zhao E., Tao K., Li R. MicroRNA-152 regulates immune response via targeting B7-H1 in gastric carcinoma. *Oncotarget*. **2017**; 8: 28125-28134.

S33. Weissman R, Diamond EL, Haroche J, Durham BH, Cohen F, Buthorn J, Amoura Z, Emile JF, Mazor RD, Shomron N, Abdel-Wahab OI, Shpilberg O, Hershkovitz-Rokah O. MicroRNA-15a-5p acts as a tumor suppressor in histiocytosis by mediating CXCL10-ERK-LIN28a-let-7 axis. *Leukemia*. **2022** Apr;36(4):1139-1149

S34. Eyileten, C., Sharif, L., Wicik, Z., Jakubik, D., Jarosz-Popek, J., Soplinska, A., Postula, M., Czlonkowska, A., Kaplon-Cieslicka, A., & Mirowska-Guzel, D. The Relation of the Brain-Derived Neurotrophic Factor with MicroRNAs in Neurodegenerative Diseases and Ischemic Stroke. *Molecular neurobiology*, **2021**, 58(1), 329–347. https://doi.org/10.1007/s12035-020-02101-2

S35. Saba R, Störchel PH, Aksoy-Aksel A, Kepura F, Lippi G, Plant TD, Schratt GM. Dopamine-regulated microRNA MiR-181a controls GluA2 surface expression in hippocampal neurons. *Mol Cell Biol*. **2012** Feb;32(3):619-32.

S36. Ding H, Yao J, Xie H, Wang C, Chen J, Wei K, Ji Y, Liu L. MicroRNA-195-5p Downregulation Inhibits Endothelial Mesenchymal Transition and Myocardial Fibrosis in Diabetic Cardiomyopathy by Targeting Smad7 and Inhibiting Transforming Growth Factor Beta 1-Smads-Snail Pathway. *Front Physiol*. **2021** Sep 30;12:709123.

S37. Arzhanov I, Sintakova K, Romanyuk N. The Role of miR-20 in Health and Disease of the Central Nervous System. *Cells*. **2022** May 3;11(9):1525.

S38. Guan C, Luan L, Li J, Yang L. MiR-212-3p improves rat functional recovery and inhibits neurocyte apoptosis in spinal cord injury models via PTEN downregulation-mediated activation of AKT/mTOR pathway. *Brain Res*. **2021** Oct 1;1768:147576.

S39. Oladejo AO, Li Y, Imam BH, Ma X, Shen W, Wu X, Jiang W, Yang J, Lv Y, Ding X, Wang S, Yan Z. MicroRNA miR-24-3p Mediates the Negative Regulation of Lipopolysaccharide-Induced Endometrial Inflammatory Response by Targeting TNF Receptor-Associated Factor 6 (TRAF6). *J Inflamm Res*. **2022** Feb 6;15:807-825.

S40. Xiao Y, Zheng S, Duan N, Li X, Wen J. MicroRNA-26b-5p alleviates cerebral ischemia-reperfusion injury in rats via inhibiting the N-myc/PTEN axis by downregulating KLF10 expression. *Hum Exp Toxicol*. **2021** Aug;40(8):1250-1262.

S41. Harati R, Hammad S, Tlili A, Mahfood M, Mabondzo A, Hamoudi R. miR-27a-3p regulates expression of intercellular junctions at the brain endothelium and controls the endothelial barrier permeability. *PLoS One*. **2022** Jan 13;17(1):e0262152.

S42. Camkurt, M.A. Blood microRNA dysregulation in schizophrenia. *Psychiatry and Clinical Psychopharmacology* **2015**; 25: S112-S112 2015. https://psychiatry-psychopharmacology.com/en/blood-microrna-dysregulation-in-schizophrenia-13649.

S43. Wang J, Zhu M, Ye L, Chen C, She J, Song Y. MiR-29b-3p promotes particulate matter-induced inflammatory responses by regulating the C1QTNF6/AMPK pathway. *Aging* (Albany NY). **2020** Jan 18;12(2):1141-1158.

S44. Hu Z, Cai M, Zhang Y, Tao L, Guo R. miR-29c-3p inhibits autophagy and cisplatin resistance in ovarian cancer by regulating FOXP1/ATG14 pathway. Cell Cycle. 2020 Jan;19(2):193-206.

S45. Yuan JN, Hong Y, Ma ZL, Pang RP, Lei QQ, Lv XF, Zhou JG, Huang H, Zhang TT. MiR-302a Limits Vascular Inflammation by Suppressing Nuclear Factor-κ B Pathway in Endothelial Cells. Front Cell Dev Biol. 2021 Aug 2;9:682574.

S46. Yasukawa K, Kinoshita D, Yaku K, Nakagawa T, Koshiba T. The microRNAs miR-302b and miR-372 regulate mitochondrial metabolism via the SLC25A12 transporter, which controls MAVS-mediated antiviral innate immunity. J Biol Chem. 2020 Jan 10;295(2):444-457.

S47. Li J, Salvador AM, Li G, Valkov N, Ziegler O, Yeri A, Yang Xiao C, Meechoovet B, Alsop E, Rodosthenous RS, Kundu P, Huan T, Levy D, Tigges J, Pico AR, Ghiran I, Silverman MG, Meng X, Kitchen R, Xu J, Van Keuren-Jensen K, Shah R, Xiao J, Das S. Mir-30d Regulates Cardiac Remodeling by Intracellular and Paracrine Signaling. Circ Res. 2021 Jan 8;128(1):e1-e23.

S48. Zhang R, Shi H, Ren F, Feng W, Cao Y, Li G, Liu Z, Ji P, Zhang M. MicroRNA-338-3p suppresses ovarian cancer cells growth and metastasis: implication of Wnt/catenin beta and MEK/ERK signaling pathways. J Exp Clin Cancer Res. 2019 Dec 16;38(1):494.

S49. Fletcher CE, Deng L, Orafidiya F, Yuan W, Lorentzen MPGS, Cyran OW, Varela-Carver A, Constantin TA, Leach DA, Dobbs FM, Figueiredo I, Gurel B, Parkes E, Bogdan D, Pereira RR, Zhao SG, Neeb A, Issa F, Hester J, Kudo H, Liu Y, Philippou Y, Bristow R, Knudsen K, Bryant RJ, Feng FY, Reed SH, Mills IG, de Bono J, Bevan CL. A non-coding RNA balancing act: miR-346-induced DNA damage is limited by the long non-coding RNA NORAD in prostate cancer. Mol Cancer. 2022 Mar 22;21(1):82.

S50. Zhao C, Zhou Y, Ran Q, Yao Y, Zhang H, Ju J, Yang T, Zhang W, Yu X, He S. MicroRNA-381-3p Functions as a Dual Suppressor of Apoptosis and Necroptosis and Promotes Proliferation of Renal Cancer Cells. Front Cell Dev Biol. 2020 Apr 28;8:290.

S51. Wan L, Zhu L, Xu J, Lu B, Yang Y, Liu F, Wang Z. MicroRNA-409-3p functions as a tumor suppressor in human lung adenocarcinoma by targeting c-Met. Cell Physiol Biochem. 2014;34(4):1273-90.

S52. Deng X, Zuo M, Pei Z, Xie Y, Yang Z, Zhang Z, Jiang M, Kuang D. MicroRNA-455-5p Contributes to Cholangiocarcinoma Growth and Mediates Galangin's Anti-Tumor Effects. J Cancer. 2021 Jun 4;12(15):4710-4721.

S53. Zhao Z, Shuang T, Gao Y, Lu F, Zhang J, He W, Qu L, Chen B, Hao Q. Targeted delivery of exosomal miR-484 reprograms tumor vasculature for chemotherapy sensitization. Cancer Lett. 2022 Apr 1;530:45-58.

S54. Gao F, Wu H, Wang R, Guo Y, Zhang Z, Wang T, Zhang G, Liu C, Liu J. MicroRNA-485-5p suppresses the proliferation, migration and invasion of small cell lung cancer cells by targeting flotillin-2. Bioengineered. 2019 Dec;10(1):1-12.

S55. Ma M, He M, Jiang Q, Yan Y, Guan S, Zhang J, Yu Z, Chen Q, Sun M, Yao W, Zhao H, Jin F, Wei M. MiR-487a Promotes TGF-β1-induced EMT, the Migration and Invasion of Breast Cancer Cells by Directly Targeting MAGI2. Int J Biol Sci. 2016 Feb 5;12(4):397-408.

S56. Gao W, Zheng W, Sun Y, Xu T. microRNA-489 negatively modulates RIG-I signaling pathway via targeting TRAF6 in miiuy croaker after poly(I:C) stimulation. Fish Shellfish Immunol. 2021 Jun;113:61-68.

S57. Ravegnini G, De Leo A, Coada C, Gorini F, de Biase D, Ceccarelli C, Dondi G, Tesei M, De Crescenzo E, Santini D, Corradini AG, Tallini G, Hrelia P, De Iaco P, Angelini S, Perrone AM. Identification of miR-499a-5p as a Potential Novel Biomarker for Risk Stratification in Endometrial Cancer. Front Oncol. 2021 Oct 29;11:757678.

S58. Chen S, Zhou H, Zhang B, Hu Q. Exosomal miR-512-3p derived from mesenchymal stem cells inhibits oxidized low-density lipoprotein-induced vascular endothelial cells dysfunction via regulating Keap1. J Biochem Mol Toxicol. 2021 Jun;35(6):1-11.

S59. Liu M, Wang Y, Lu H, Wang H, Shi X, Shao X, Li YX, Zhao Y, Wang YL. miR-518b Enhances Human Trophoblast Cell Proliferation Through Targeting Rap1b and Activating Ras-MAPK Signal. Front Endocrinol (Lausanne). 2018 Mar 15;9:100.

S60. Augenlicht A, Saiselet M, Decaussin-Petrucci M, Andry G, Dumont JE, Maenhaut C. MiR-7-5p inhibits thyroid cell proliferation by targeting the EGFR/MAPK and IRS2/PI3K signaling pathways. Oncotarget. 2021 Aug 3;12(16):1587-1599.

S61. Zhen J, Zhang H, Dong H, Tong X. miR-9-3p inhibits glioma cell proliferation and apoptosis by directly targeting FOXG1. Oncol Lett. 2020 Aug;20(2):2007-2015.

S62. Jinghua H, Qinghua Z, Chenchen C, Lili C, Xiao X, Yunfei W, Zhengzhe A, Changxiu L, Hui H. MicroRNA miR-92a-3p regulates breast cancer cell proliferation and metastasis via regulating B-cell translocation gene 2 (BTG2). Bioengineered. 2021 Dec;12(1):2033-2044.

S63. Bhattacharyya, P., Biswas, A., & Biswas, S. C. Brain-enriched miR-128: Reduced in exosomes from Parkinson's patient plasma, improves synaptic integrity, and prevents 6-OHDA mediated neuronal apoptosis. *Frontiers in cellular neuroscience*, **2023**, 16, 1037903.

S64. Koh HS, Lee S, Lee HJ, Min JW, Iwatsubo T, Teunissen CE, Cho HJ, Ryu JH. Targeting MicroRNA-485-3p Blocks Alzheimer's Disease Progression. Int J Mol Sci. 2021 Dec 4;22(23):13136.

S65. Yoon S, Han E, Choi YC, Kee H, Jeong Y, Yoon J, Baek K. Inhibition of cell proliferation and migration by miR-509-3p that targets CDK2, Rac1, and PIK3C2A. Mol Cells. 2014 Apr;37(4):314-21.

S66. Regis S, Dondero A, Spaggiari GM, Serra M, Caliendo F, Bottino C, Castriconi R. miR-24-3p down-regulates the expression of the apoptotic factors FasL and BIM in human natural killer cells. Cell Signal. 2022 Oct;98:110415.

S67. Li L, Zhang X, Lin Y, Ren X, Xie T, Lin J, Wu S, Ye Q. Let-7b-5p inhibits breast cancer cell growth and metastasis via repression of hexokinase 2-mediated aerobic glycolysis. Cell Death Discov. 2023 Apr 5;9(1):114.

S68. Liu JH, Li C, Cao L, Zhang CH, Zhang ZH. Cucurbitacin B regulates lung cancer cell proliferation and apoptosis via inhibiting the IL-6/STAT3 pathway through the lncRNA XIST/miR-let-7c axis. Pharm Biol. 2022 Dec;60(1):154-162.

S69. Chen YN, Ren CC, Yang L, Nai MM, Xu YM, Zhang F, Liu Y. MicroRNA let 7d 5p rescues ovarian cancer cell apoptosis and restores chemosensitivity by regulating the p53 signaling pathway via HMGA1. Int J Oncol. 2019 May;54(5):1771-1784.

S70. Chen W, Lin G, Yao Y, Chen J, Shui H, Yang Q, Wang X, Weng X, Sun L, Chen F, Yang S, Yang Y, Zhou Y. MicroRNA hsa-let-7e-5p as a potential prognosis marker for rectal carcinoma with liver metastases. Oncol Lett. 2018 May;15(5):6913-6924.

71. Zhang K, Yang R, Chen J, Qi E, Zhou S, Wang Y, Fu Q, Chen R, Fang X. Let-7i-5p Regulation of Cell Morphology and Migration Through Distinct Signaling Pathways in Normal and Pathogenic Urethral Fibroblasts. Front Bioeng Biotechnol. 2020 May 14;8:428.

S72. Xue P, Huang S, Han X, Zhang C, Yang L, Xiao W, Fu J, Li H, Zhou Y. Exosomal miR-101-3p and miR-423-5p inhibit medulloblastoma tumorigenesis through targeting FOXP4 and EZH2. Cell Death Differ. 2022 Jan;29(1):82-95.

S73. Sagar SK. miR-106b as an emerging therapeutic target in cancer. Genes Dis. 2021 Feb 12;9(4):889-899.

S74. Ahonen MA, Haridas PAN, Mysore R, Wabitsch M, Fischer-Posovszky P, Olkkonen VM. miR-107 inhibits CDK6 expression, differentiation, and lipid storage in human adipocytes. Mol Cell Endocrinol. 2019 Jan 5;479:110-116.

S75. Qu C, Liu X, Guo Y, Fo Y, Chen X, Zhou J, Yang B. MiR-128-3p inhibits vascular smooth muscle cell proliferation and migration by repressing FOXO4/MMP9 signaling pathway. Mol Med. 2020 Nov 25;26(1):116.

S76. Khalili N, Nouri-Vaskeh M, Hasanpour Segherlou Z, Baghbanzadeh A, Halimi M, Rezaee H, Baradaran B. Diagnostic, prognostic, and therapeutic significance of miR-139-5p in cancers. Life Sci. 2020 Sep 1;256:117865.

S77. Wang Z, Liu F, Wei M, Qiu Y, Ma C, Shen L, Huang Y. Chronic constriction injury-induced microRNA-146a-5p alleviates neuropathic pain through suppression of IRAK1/TRAF6 signaling pathway. J Neuroinflammation. 2018 Jun 9;15(1):179.

S78. Maurel, O. M., Torrisi, S. A., Barbagallo, C., Purrello, M., Salomone, S., Drago, F., Ragusa, M., & Leggio, G. M. Dysregulation of miR-15a-5p, miR-497a-5p and miR-511-5p Is Associated with Modulation of BDNF and FKBP5 in Brain Areas of PTSD-Related Susceptible and Resilient Mice. *International journal of molecular sciences*, **2021**, 22(10), 5157.

S79. Liu F, Di Wang X. miR-150-5p represses TP53 tumor suppressor gene to promote proliferation of colon adenocarcinoma. Sci Rep. 2019 May 1;9(1):6740.

S80. Li B, Xia Y, Lv J, Wang W, Xuan Z, Chen C, Jiang T, Fang L, Wang L, Li Z, He Z, Li Q, Xie L, Qiu S, Zhang L, Zhang D, Xu H, Xu Z. miR-151a-3p-rich small extracellular vesicles derived from gastric cancer accelerate liver metastasis via initiating a hepatic stemness-enhancing niche. Oncogene. 2021 Oct;40(43):6180-6194.

S81. Su Y, Yuan J, Zhang F, Lei Q, Zhang T, Li K, Guo J, Hong Y, Bu G, Lv X, Liang S, Ou J, Zhou J, Luo B, Shang J. MicroRNA-181a-5p and microRNA-181a-3p cooperatively restrict vascular inflammation and atherosclerosis. Cell Death Dis. 2019 May 7;10(5):365.

S82. Yu Y, Cai W, Xu Y, Zuo W. Down-regulation of miR-19b-3p enhances IGF-1 expression to induce osteoblast differentiation and improve osteoporosis. Cell Mol Biol (Noisy-le-grand). 2022 May 22;68(1):160-168.

S83. Han J, Hu J, Sun F, Bian H, Tang B, Fang X. MicroRNA-20a-5p suppresses tumor angiogenesis of non-small cell lung cancer through RRM2-mediated PI3K/Akt signaling pathway. Mol Cell Biochem. 2021 Feb;476(2):689-698.

S84. Gorur A, Bayraktar R, Ivan C, Mokhlis HA, Bayraktar E, Kahraman N, Karakas D, Karamil S, Kabil NN, Kanlikilicer P, Aslan B, Tamer L, Wang Z, Cristini V, Lopez-Berestein G, Calin G, Ozpolat B. ncRNA therapy with miRNA-22-3p suppresses the growth of triple-negative breast cancer. Mol Ther Nucleic Acids. 2021 Jan 20;23:930-943.

S85. Zhang, H., Liang, J., & Chen, N. (2022). The Potential Role of miRNA-Regulated Autophagy in Alzheimer's Disease. International Journal of Molecular Sciences, 23(14).

S86. Zhou A, Pan H, Sun D, Xu H, Zhang C, Chen X, Li L, Wang T. miR-26b-5p Inhibits the Proliferation, Migration and Invasion of Human Papillary Thyroid Cancer in a β-Catenin-Dependent Manner. Onco Targets Ther. 2020 Feb 21;13:1593-1603.

S87. Duwe L, Munoz-Garrido P, Lewinska M, Lafuente-Barquero J, Satriano L, Høgdall D, Taranta A, Nielsen BS, Ghazal A, Matter MS, Banales JM, Aldana BI, Gao YT, Marquardt JU, Roberts LR, Oliveira RC, Koshiol J, O'Rourke CJ, Andersen JB. MicroRNA-27a-3p targets FoxO signalling to induce tumour-like phenotypes in bile duct cells. J Hepatol. 2023 Feb;78(2):364-375.

S88. Lv Y, Yang H, Ma X, Wu G. Strand-specific miR-28-3p and miR-28-5p have differential effects on nasopharyngeal cancer cells proliferation, apoptosis, migration and invasion. Cancer Cell Int. 2019 Jul 19;19:187.

S89. Sun H, Zhong D, Wang C, Sun Y, Zhao J, Li G. MiR-298 Exacerbates Ischemia/Reperfusion Injury Following Ischemic Stroke by Targeting Act1. Cell Physiol Biochem. 2018;48(2):528-539.

S90. Zhang K, Han X, Hu W, Su C, He B. miR-29a-3p inhibits the malignant characteristics of non-small cell lung cancer cells by reducing the activity of the Wnt/β-catenin signaling pathway. Oncol Lett. 2022 Sep 12;24(4):379.

S91. Ma, X., Yun, H. J., Elkin, K., Guo, Y., Ding, Y., & Li, G. (2022). MicroRNA-29b Suppresses Inflammation and Protects Blood-Brain Barrier Integrity in Ischemic Stroke. *Mediators of inflammation,* **2022**, 1755416. https://doi.org/10.1155/2022/1755416

S92. Taghehchian, N., Lotfi, M., Zangouei, A. S., Akhlaghipour, I., & Moghbeli, M. MicroRNAs as the critical regulators of Forkhead box protein family during gynecological and breast tumor progression and metastasis. *European journal of medical research*, **2023**, 28(1), 330. https://doi.org/10.1186/s40001-023-01329-7

S93. Chuang, Y.-T.; Tang, J.-Y.; Shiau, J.-P.; Yen, C.-Y.; Chang, F.-R.; Yang, K.-H.; Hou, M.-F.; Farooqi, A.A.; Chang, H.-W. Modulating Effects of Cancer-Derived Exosomal miRNAs and Exosomal Processing by Natural Products. *Cancers* **2023**, 15, 318. https://doi.org/10.3390/cancers15010318.

S94. Du H, Yin Z, Zhao Y, Li H, Dai B, Fan J, He M, Nie X, Wang CY, Wang DW, Chen C. miR-320a induces pancreatic β cells dysfunction in diabetes by inhibiting MafF. Mol Ther Nucleic Acids. 2021 Aug 26;26:444-457.

S95. Chen KC, Hsi E, Hu CY, Chou WW, Liang CL, Juo SH. MicroRNA-328 may influence myopia development by mediating the PAX6 gene. Invest Ophthalmol Vis Sci. 2012 May 31;53(6):2732-9.

S96. Wang Q, Cai J, Cai XH, Chen L. miR-346 regulates osteogenic differentiation of human bone marrow-derived mesenchymal stem cells by targeting the Wnt/β-catenin pathway. PLoS One. 2013 Sep 4;8(9):e72266.

S97. Zhang L, Liao Y, Tang L. MicroRNA-34 family: a potential tumor suppressor and therapeutic candidate in cancer. J Exp Clin Cancer Res. 2019 Feb 4;38(1):53.

98. SLi H, Xue Y, Ma J, Shao L, Wang D, Zheng J, Liu X, Yang C, He Q, Ruan X, Li Z, Liu Y. SNHG1 promotes malignant biological behaviors of glioma cells via microRNA-154-5p/miR-376b-3p- FOXP2- KDM5B participating positive feedback loop. J Exp Clin Cancer Res. 2019 Feb 6;38(1):59.

S99. Jiang H, Su Z, Hu W, Yuan X, Yu T, Yang J, Xiao X, Zheng S, Lin B. miR-433 Inhibits Glioblastoma Progression by Suppressing the PI3K/Akt Signaling Pathway Through Direct Targeting of ERBB4. OMICS. 2023 May;27(5):215-226.

S100. Gao J, Dai C, Yu X, Yin XB, Zhou F. microRNA-485-5p inhibits the progression of hepatocellular carcinoma through blocking the WBP2/Wnt signaling pathway. Cell Signal. 2020 Feb;66:109466.

S101. Yang Y, Li H, He Z, Xie D, Ni J, Lin X. MicroRNA-488-3p inhibits proliferation and induces apoptosis by targeting ZBTB2 in esophageal squamous cell carcinoma. J Cell Biochem. 2019 Nov;120(11):18702-18713.

S102. Yang L, Xu X, Chen Z, Zhang Y, Chen H, Wang X. miR-511-3p promotes hepatic sinusoidal obstruction syndrome by activating hedgehog pathway via targeting Ptch1. Am J Physiol Gastrointest Liver Physiol. 2021 Sep 1;321(3):G344-G354.

S103. Li X, He L, Yue Q, Lu J, Kang N, Xu X, Wang H, Zhang H. MiR-9-5p promotes MSC migration by activating β-catenin signaling pathway. Am J Physiol Cell Physiol. 2017 Jul 1;313(1):C80-C93.

S104. Yang, B., Feng, X., Liu, H. et al. High-metastatic cancer cells derived exosomal miR92a-3p promotes epithelial-mesenchymal transition and metastasis of low-metastatic cancer cells by regulating PTEN/Akt pathway in hepatocellular carcinoma. *Oncogene*, **2020**, 39, 6529–6543.

S105. Yang M, Xiao R, Wang X, Xiong Y, Duan Z, Li D, Kan Q. MiR-93-5p regulates tumorigenesis and tumor immunity by targeting PD-L1/CCND1 in breast cancer. Ann Transl Med. 2022 Feb;10(4):203.

S106. Du Y, Shi X, Li J, Jia Y. MicroRNA-98-5p inhibits human mesangial cell proliferation and TNF-α and IL-6 secretion by targeting BTB and CNC homology 1. Exp Ther Med. 2021 Dec;22(6):1436.

S107. Pajares, M. J., Alemany-Cosme, E., Goñi, S., Bandres, E., Palanca-Ballester, C., & Sandoval, J. (2021). Epigenetic Regulation of microRNAs in Cancer: Shortening the Distance from Bench to Bedside. International journal of molecular sciences, 22(14), 7350..

S108. Geng, L., Zhang, T., Liu, W., & Chen, Y. Inhibition of miR-128 Abates Aβ-Mediated Cytotoxicity by Targeting PPAR-γ via NF-κB Inactivation in Primary Mouse Cortical Neurons and Neuro2a Cells. *Yonsei medical journal*, **2018**, 59(9), 1096–1106.

S109. Huang, P., Wei, S., Luo, M., Tang, Z., Lin, Q., Wang, X., Luo, M., He, Y., Wang, C., Wei, D., Xia, C., & Xu, J. MiR-139-5p has an antidepressant-like effect by targeting phosphodiesterase 4D to activate the cAMP/PKA/CREB signaling pathway. Annals of translational medicine, 2021, 9(20), 1594.

S110. Liao, Z., Zheng, R., & Shao, G. Mechanisms and application strategies of miRNA-146a regulating inflammation and fibrosis at molecular and cellular levels (Review). *International journal of molecular medicine*, **2023**, 51(1), 7.

S111. Li W, Wu L, Sun Q, Yang Q, Xue J, Shi M, Tang H, Zhang J, Liu Q. MicroRNA-191 blocking the translocation of GLUT4 is involved in arsenite-induced hepatic insulin resistance through inhibiting the IRS1/AKT pathway. Ecotoxicol Environ Saf. 2021 Jun 1;215:112130.

S112. He J, Han Z, An Z, Li Y, Xie X, Zhou J, He S, Lv Y, He M, Qu H, Liu G, Li Y. The miR-203a Regulatory Network Affects the Proliferation of Chronic Myeloid Leukemia K562 Cells. Front Cell Dev Biol. 2021 Feb 15;9:616711.

S113. Zhu, X. A., Gao, L. F., Zhang, Z. G., & Xiang, D. K. (2019). Down-regulation of miR-320 exerts protective effects on myocardial I-R injury via facilitating Nrf2 expression. European review for medical and pharmacological sciences, 23(4), 1730–1741..

S114. Cui H, Song R, Wu J, Wang W, Chen X, Yin J. MicroRNA-337 regulates the PI3K/AKT and Wnt/β-catenin signaling pathways to inhibit hepatocellular carcinoma progression by targeting high-mobility group AT-hook 2. Am J Cancer Res. 2018 Mar 1;8(3):405-421.

S115. Tian W, Yang X, Yang H, Lv M, Sun X, Zhou B. Correction: Exosomal miR-338-3p suppresses non-small-cell lung cancer cells metastasis by inhibiting CHL1 through the MAPK signaling pathway. Cell Death Dis. 2022 May 19;13(5):473.

S116. Gao JR, Shi MM, Jiang H, Zhu XL, Wei LB, Qin XJ. MicroRNA-339-5p inhibits lipopolysaccharide-induced rat mesangial cells by regulating the Syk/Ras/c-Fos pathway. Naunyn Schmiedebergs Arch Pharmacol. 2022 Sep;395(9):1075-1085.

S117. Zhang S, Liu L, Lv Z, Li Q, Gong W, Wu H. MicroRNA-342-3p Inhibits the Proliferation, Migration, and Invasion of Osteosarcoma Cells by Targeting Astrocyte-Elevated Gene-1 (AEG-1). Oncol Res. 2017 Nov 2;25(9):1505-1515.

S118. Li Z, Zhao H, Chu S, Liu X, Qu X, Li J, Liu D, Li H. miR-124-3p promotes BMSC osteogenesis via suppressing the GSK-3β/β-catenin signaling pathway in diabetic osteoporosis rats. In Vitro Cell Dev Biol Anim. 2020 Oct;56(9):723-734.

S119. Fang H, Li HF, Pan Q, Jin HL, Yang M, Wang RR, Wang QY, Zhang JP. MiR-132-3p Modulates MEKK3-Dependent NF-κB and p38/JNK Signaling Pathways to Alleviate Spinal Cord Ischemia-Reperfusion Injury by Hindering M1 Polarization of Macrophages. Front Cell Dev Biol. 2021 Feb 11;9:570451.

S120. Si YJ, Ren QH, Bi L. miR-135b-5p regulates human mesenchymal stem cell osteogenic differentiation by facilitating the Hippo signaling pathway. Int J Clin Exp Pathol. 2017 Jul 1;10(7):7767-7775.

S121. Deng X, Chu X, Wang P, Ma X, Wei C, Sun C, Yang J, Li Y. MicroRNA-29a-3p Reduces TNFα-Induced Endothelial Dysfunction by Targeting Tumor Necrosis Factor Receptor 1. Mol Ther Nucleic Acids. 2019 Dec 6;18:903-915.

S122. Lian, H., Zhong, X. S., Xiao, Y., Sun, Z., Shen, Y., Zhao, K., Ma, X., Li, Y., Niu, Q., Liu, M., Powell, D. W., Liu, C., & Li, Q. Exosomal miR-29b of Gut Origin in Patients With Ulcerative Colitis Suppresses Heart Brain-Derived Neurotrophic Factor. *Frontiers in molecular biosciences,* **2022**, 9, 759689.

S123. Liu J, Zuo X, Han J, Dai Q, Xu H, Liu Y, Cui S. MiR-9-5p inhibits mitochondrial damage and oxidative stress in AD cell models by targeting GSK-3β. Biosci Biotechnol Biochem. 2020 Nov;84(11):2273-2280.

S124. Sim, S. E., Lim, C. S., Kim, J. I., Seo, D., Chun, H., Yu, N. K., Lee, J., Kang, S. J., Ko, H. G., Choi, J. H., Kim, T., Jang, E. H., Han, J., Bak, M. S., Park, J. E., Jang, D. J., Baek, D., Lee, Y. S., & Kaang, B. K. The Brain-Enriched MicroRNA miR-9-3p Regulates Synaptic Plasticity and Memory. The Journal of neuroscience: the official journal of the Society for Neuroscience, 2016, 36(33), 8641–8652.

S125. Lu XC, Zheng JY, Tang LJ, Huang BS, Li K, Tao Y, Yu W, Zhu RL, Li S, Li LX. MiR-133b Promotes neurite outgrowth by targeting RhoA expression. Cell Physiol Biochem. 2015;35(1):246-58.

S126. Xu, C., Bai, Q., Wang, C., Meng, Q., Gu, Y., Wang, Q., Xu, W., Han, Y., Qin, Y., Jia, S., Zhang, J., Xu, J., Li, J., Chen, M., & Wang, F. miR-433 Inhibits Neuronal Growth and Promotes Autophagy in Mouse Hippocampal HT-22 Cell Line. *Frontiers in pharmacology*, **2022**, 11, 536913..

S127. Fernández-de Frutos, M., Galán-Chilet, I., Goedeke, L., Kim, B., Pardo-Marqués, V., Pérez-García, A., Herrero, J. I., Fernández-Hernando, C., Kim, J., & Ramírez, C. M. MicroRNA 7 Impairs Insulin Signaling and Regulates Aβ Levels through Posttranscriptional Regulation of the Insulin Receptor Substrate 2, Insulin Receptor, Insulin-Degrading Enzyme, and Liver X Receptor Pathway. *Molecular and cellular biology*, **2019**, 39(22), e00170-19.

S128. Liu, N., Yang, C., Gao, A., Sun, M., & Lv, D. MiR-101: An Important Regulator of Gene Expression and Tumor Ecosystem. Cancers, 2022, 14(23), 5861. https://doi.org/10.3390/cancers14235861.

S129. Al-hasso, I. K. Q., Al-Derzi, A. R., Abbas, A. A. H., Gorial, F. I., & Alnuimi, A. S. Role of circulating miRNA-130b-3p and TGF-β 1cytokine in patients with systemic lupus erythematosus. Gene Reports, 2022, 26, 101476.

S130. Zhao Y, Li A. miR-19b-3p relieves intervertebral disc degeneration through modulating PTEN/PI3K/Akt/mTOR signaling pathway. *Aging* (Albany NY). **2021** Sep 23;13(18):22459-22473. doi: 10.18632/aging.203553. Epub 2021 Sep 23.
